# Metabolites Secreted by a Plant-Growth-Promoting *Pantoea agglomerans* Strain Improved Rooting of *Pyrus communis* L. cv Dar Gazi Cuttings

**DOI:** 10.3389/fmicb.2020.539359

**Published:** 2020-10-14

**Authors:** Francesca Luziatelli, Lorenzo Gatti, Anna Grazia Ficca, Gabriele Medori, Cristian Silvestri, Francesca Melini, Rosario Muleo, Maurizio Ruzzi

**Affiliations:** ^1^Department for Innovation in Biological, Agrofood and Forest Systems (DIBAF), University of Tuscia, Viterbo, Italy; ^2^Department of Agricultural and Forest Sciences (DAFNE), University of Tuscia, Viterbo, Italy; ^3^CREA Research Centre for Food and Nutrition, Rome, Italy

**Keywords:** plant-growth-promoting rhizobacteria, auxin, biostimulants, plant tissue culture, auxin-regulated genes, adventitious root, micropropagation

## Abstract

Strains belonging to *Pantoea agglomerans* species are known for their ability to produce metabolites that can act in synergy with auxins to induce the adventitious root (AR) formation. The latter is critically important in the agamic propagation of several woody species, including pear (*Pyrus communis* L.), playing a considerable role in the commercial nursery farms including those using micropropagation techniques. When grown on a medium amended with tryptophan, the plant-growth-promoting (PGP) strain *P. agglomerans* C1 produces a cocktail of auxin and auxin-like molecules that can be utilized as biostimulants to improve the rooting of vegetable (*Solanum lycopersicum* L.) and woody crop species (*Prunus* rootstock GF/677 and hazelnut). In this study, we evaluated the morphological and molecular responses induced by strain C1 exometabolites in microcuttings of *P. communis* L. cv Dar Gazi and the potential benefits arising from their application. Results showed that exometabolites by *P. agglomerans* C1 induced a direct and earlier emergence of roots from stem tissues and determined modifications of root morphological parameters and root architecture compared to plants treated with the synthetic hormone indole-3-butyric acid (IBA). Transcription analysis revealed differences in the temporal expression pattern of *ARF17* gene when IBA and C1 exometabolites were used alone, while together they also determined changes in the expression pattern of other key auxin-regulated plant genes. These results suggest that the phenotypic and molecular changes triggered by *P. agglomerans* C1 are dependent on different stimulatory and inhibitory effects that auxin-like molecules and other metabolites secreted by this strain have on the gene regulatory network of the plant. This evidence supports the hypothesis that the strategies used to harness the metabolic potential of PGP bacteria are key factors in obtaining novel biostimulants for sustainable agriculture. Our results demonstrate that metabolites secreted by strain C1 can be successfully used to increase the efficiency of micropropagation of pear through tissue culture techniques.

## Introduction

A growing body of research demonstrated that cells and metabolites from plant-growth-promoting (PGP) bacteria and fungi can be successfully used as biostimulants to promote plant growth, enhancing nutrient uptake and photosynthetic activity, as well as to increase crop quality and plant resistance to both biotic and abiotic stresses (Wu and Zou, 2009; Glick, 2014; Ruzzi and Aroca, 2015; Colla et al., 2017; Rouphael and Colla, 2018; Woo and Pepe, 2018). Due to the increasing demand of high-quality planting materials through agamic propagation and, in particular, tissue culture techniques (Twaij et al., 2020), there is rising interest in the use of microbial and nonmicrobial biostimulants in the improvement of micropropagation methods for industrial, ornamental, and food vegetable and fruit crops (Campobenedetto et al., 2020; Cardarelli et al., 2020; Cirillo et al., 2020; de Azevedo et al., 2020; Dong et al., 2020; Gulzar et al. 2020; Luziatelli et al. 2020b; Umanzor et al., 2020). In this respect, beneficial microorganisms able to synthesize or metabolize plant hormones are of particular importance because they can modulate the balance between plant growth and defense against stresses and pathogens, by changing the concentration of specific phytohormones (Tsukanova et al., 2017; Saia et al., 2019, 2020; Rouphael et al., 2020).

In the context of plant tissue culture, one of the crucial processes in the vegetative propagation of many woody species is the formation of adventitious rooting (AR) from the stem, which involves the changing of the destiny of stem cells near the wound region which leads to the organization of new radical meristem (Garrido et al., 2002). For many woody species, including pear (one of the world’s most important fruit crops; FAOSTAT, 2018^1^), this process is critical even in *in vitro* culture systems, making difficult the production of many plants on an industrial propagation scale and maintenance of the genetic identity of elite genotypes. Among many endogenous and environmental factors, auxins, such as indole-3-acetic acid (IAA), play a pivotal role for priming, initiation, and establishment of adventitious roots (Wiesman et al., 1988; Li et al., 2009; Pacurar et al., 2014; Druege et al., 2019) and in the regulation of root system architecture in response to internal signals and environmental stimuli (Overvoorde et al., 2010; Semeradova et al., 2020; Waidmann et al., 2020).

Plants synthesize auxins in the shoot apical meristem of young leaves, and then these molecules are transported and concentrated at the base of cutting through basipetal polar transport (Ahkami et al., 2013). Generally, the concentration of endogenous auxins at the base of cutting does not sufficiently induce AR; exogenous auxin is thus added to the culture medium used for *in vitro* culture systems to reach the optimal gradient. Many synthetic auxins are then used to induce roots on the shoot, and the use of these compounds in commercial plant propagation is commonly implemented (Kurepin et al., 2011; Reed et al., 2012). Each auxin has a different ability to induce AR, which can be attributed to the differences in the auxin receptors involved in the rhizogenic process, as well as to the interaction with other molecules and environmental factors that can interfere with the process (de Klerk et al., 1999; Tereso et al., 2008; Iacona and Muleo, 2010; da Costa et al., 2013; Daud et al., 2013). It is also known that plants exhibit different dose-dependent responses to exogenous auxin gradients: low concentrations of IAA can stimulate primary root elongation, whereas high IAA levels have an opposite effect (Thimann, 1939; Enders and Strader, 2015).

Several bacterial species, including *Pantoea agglomerans*, can produce IAA and auxin-related compounds that have great physiological relevance in bacteria-plant interactions and determine modifications both in the root system architecture and in the structure of root tissues (Spaepen, 2015). Production of bacterial auxins varies among different strains and is influenced by strain growth conditions, as well as by precursor (tryptophan) availability (Duca et al., 2014). In addition, IAA and IAA-related compounds are frequently produced together with other plant growth regulators that can enhance the growth stimulatory effect of microbial phytohormones (Stringlis et al., 2018).

*P. agglomerans* is a rod-shaped, non-spore-forming, Gram-negative bacterium that is found in soil and is adapted to live in association with plants (Walterson and Stavrinides, 2015; Zhang and Qiu, 2015). Certain strains belonging to this species are agronomically relevant for their PGP traits and biocontrol activity (Dutkiewicz et al., 2016) and for their ability to produce IAA through a highly conserved indole-3-pyruvic acid (IPyA) pathway (Luziatelli et al., 2020b). *P. agglomerans* strain C1 was previously isolated from the phyllosphere of lettuce plants (*Lactuca sativa* L.) treated with plant-derived protein hydrolysates (Luziatelli et al., 2016). The strain was characterized for its ability to solubilize phosphate, produce IAA, and produce siderophores for inhibiting plant pathogens (Luziatelli et al., 2019a, 2020a). Recently, the whole genome of *P. agglomerans* C1 was sequenced, providing insights into heavy metal resistance and metabolic capacities of this strain (Luziatelli et al., 2019b, 2020a). In addition, it was demonstrated that inoculation with C1 cells improved the growth in pots of tomato (*Solanum lycopersicum* L.) and corn (*Zea mays* L.) fertilized with rock phosphates (Saia et al., 2020) and, also, that metabolites secreted by this strain can be utilized as biostimulants to improve the root surface area in cuttings of tomato (Luziatelli et al., 2020a), *Prunus* rootstocks GF/677, and *Corylus avellana* L. (Luziatelli et al., 2020b).

Considering the importance of AR induction in the agamic propagation and micropropagation of cultures for research and industrial nursery farming applications, further evaluation of the plant response to metabolites secreted by selected PGP rhizobacteria (PGPR) can provide useful insights into the rational development of new biostimulants that can contribute to achieving agriculture-related sustainable development goals. In this work, we compared the effect of indole-3-butyric acid (IBA; the most widely used synthetic auxin for cutting propagation), metabolites secreted by *P. agglomerans* C1, and a combination of both on root architectural traits and gene expression in cuttings of *Pyrus communis* L. cv Dar Gazi. In addition, we evaluated the possibility to develop an improved rooting procedure based on the use of *P. agglomerans* metabolites.

## Materials and Methods

### Bacterial Strain and Culture Conditions

*P. agglomerans* C1 was routinely cultured in Lennox LB broth (Acumedia, Baltimore, MD, USA; Miller, 1972). Shake cultures were performed in Erlenmeyer flasks at 30°C, in agitation (180 rpm). Production of IAA occurred only when tryptophan was added to the medium. Stock cultures were maintained at −80°C in LB medium amended with glycerol 20% (v/v). Bacterial metabolites used for *in vitro* rooting experiments were prepared according to a two-step culture procedure (Luziatelli et al., 2019b). Overnight pre-seed cultures were prepared by inoculating 50 ml of fresh LB medium with 0.5 ml of a glycerol stock and grown at 30°C with shaking (180 rpm). The culture was used to inoculate a 250 ml shake-flask containing 25 ml of LB supplemented with tryptophan (4 mM), with an initial optical density (OD_600_) of 0.1. Cells were grown under suboptimal temperature-agitation speed conditions (28°C and 150 rpm) to maintain auxin concentration between 200 and 350 μM IAA equivalent (IAA_equ_). After 24 h of growth, cells were separated from the exhausted medium by centrifugation (10 min at 10,000 rpm) and discharged. The supernatant was filter-sterilized (0.22 μm), the IAA concentration was determined as described below, and samples were stored at −20°C until use.

### Determination of Indole Auxins in Culture Filtrate

Auxin production was measured using Salkowski’s reagent, as described previously by Patten and Glick (2002). In brief, 1 ml of filter-sterilized (0.22 μm) supernatant was added to 2 ml of Salkowski reagent (0.5 M FeCl_3_, 35% v/v HClO_4_). The mixture was incubated for 15 min in the dark at room temperature; the development of a pink color, which was determined spectrophotometrically at 535 nm, indicated the production of indole auxins. A series of IAA standard solutions of known concentrations were prepared to set up the calibration curve.

### Plant Material

Shoot cultures of pear cultivar Dar Gazi were initiated with 5–10-mm-long shoot tips, excised from an already-established *in vitro* culture, and maintained in the proliferation medium. Proliferation medium, hormonal composition, and growth chamber conditions were reported in Abdollahi et al. (2004). Rooting experiments were run using microcuttings, about 2 cm long, cut from shoot cluster, and transferred onto rooting medium. The latter was composed of half concentrations of MS salts (Murashige and Skoog, 1962), supplemented with 20 g L^−1^ sucrose and with an appropriate amount of IBA (1.0 mM) and/or auxin-like phytohormones from *P. agglomerans* C1.

The medium was sterilized at 121°C for 20 min after the addition of 6 g L^−1^ plant agar (Duchefa Biochemie BV, The Netherlands) and pH titration to 5.7 with NaOH. To test the AR induction potential of C1 strain’s auxin-like metabolites, the medium was supplemented with the amount of exometabolites necessary to obtain an IAA_equ_ concentration of 1.0 (C1-sm) and 2.0 mM (2× C1-sm). In standard experiments, an IAA_equ_ concentration of 1.0 mM was obtained by a 250-fold dilution of the filter-sterilized supernatant of a C1 culture grown at 28°C and an agitation speed of 150 rpm. Rooting tests were carried out by using the bacterial metabolites alone or in combination with IBA 1.0 mM. Control treatments in which the medium was amended with a 250-fold dilution of LB amended with tryptophan (4 mM), with tryptophan at a final concentration of 16 mM, or with no hormonal addition were also used. All the supplements were added to the medium after sterilization immediately before pouring the plates. As culture vessels, 250 ml glass jars were used, each containing 30 ml of rooting medium.

### Experimental Design and Parameters

Experiments were performed in the growth chamber maintained at a temperature of 24 ± 1°C, by placing vessels under the darkness for a period of 4 days, after the beginning of experiments, and then moving them under the light condition of 40 μmol m^−2^ s^−1^. Five jars, each containing six explants, were used in each hormonal treatment. Measurements included the number of rooted explants, the total number of roots per shoot, and the dimension of ARs for each hormonal treatment and were detected for a 40-day period, from the beginning of experimentation. All experiments were repeated twice, and the results were the average values of both experiments.

### *Ex Vitro* Acclimatization

For *in vivo* acclimatization, rooted plantlets were transferred into plug trays (2 × 2 × 3.5 cm), containing sterile BRIL typical TYP 3 (Germany) peat substrate, and placed in a climate-controlled growth chamber, at a temperature of 25 ± 4°C, at constant photoperiod at a light intensity of 40 μmol m^−2^ s^−1^, and at a virtually constant RH (80%). Plant survival was determined after 2 months.

### RNA Extraction and Gene Expression Analyses

Total RNA was extracted starting from 450 mg of tissues collected: time point T0, before the treatment; time point T1, at the end of the dark treatment (4 days after the treatment) or, for C1-sm + IBA treatment, at the beginning of callus formation (6 days after the treatment); time point T2, after the development of the primordial roots. The apex was removed, and the tissue collected at the base of the microcutting was powdered under liquid nitrogen according to Pistelli et al. (2012). Samples were purified using a Plant RNA Purification Kit (Norgen Biotek, Thorold, ON, Canada) combined with an on-column DNase digestion with the RNase-Free DNase set (Qiagen, Hilden, Germany) to remove genomic DNA contamination. All kits were used following the manufacturer’s instructions. RNA purity was evaluated by agarose gel electrophoresis and quantified by using a QUBIT® 2.0 fluorometer (Thermo Fisher Scientific, Monza, Italy). First-strand cDNA was synthesized using the kit Tetro Reverse Transcriptase (BioLine, London, UK), following the manufacturer’s guidelines.

Real-time PCR analysis of genes was conducted using the thermal cycler LC480II® (Roche, Monza, Italy). Each reaction (10 μl) contained 6 μl of LightCycler 480 SYBR Green I Master (Roche, Monza Italy), 0.5 μM of each primer, 0.5 μl of cDNA, and 2.3 μl of PCR-grade water. The PCR was conducted using the following conditions: 95°C for 10 min and 45 cycles at 94°C for 20 s, 59°C for 30 s, and 72°C for 30 s, followed by a melting cycle from 65 to 95°C. Real-time quantitative PCR was performed using three biological replicates, with three technical replicates for each sample. A minus reverse transcriptase control was used to exclude genomic DNA contamination. Data were expressed with the 2ΔCp method (Kubista et al., 2006) using the geometric means of the actin gene as endogenous reference genes for the normalization of transcript abundance. After PCR amplification, melting analysis and gel electrophoresis visualization were run to confirm their identity and the absence of false-positive products.

### Statistical Analysis

The significance of the effects of *P. agglomerans* C1 metabolites on the AR induction and root development characters was tested by analysis of variance (ANOVA), performed by the SigmaStat 3.1 package (Systat Software Inc., San Jose, CA, USA). Means were separated by the LSD test. Data percentages were transformed to arcsine degree values before the ANOVA, when necessary.

## Results

### Auxin Production

To evaluate synergistic effects between auxins and other metabolites secreted by *P. agglomerans* C1, cells were grown in medium amended with tryptophan at suboptimal temperature (28°C) and agitation speed (150 rpm). Under these conditions, culture filtrate of strain C1 contained 286 ± 57 μmol of IAA_equ_ per liter and was used for *in vitro* experiments without extensive dilution. Under conditions of optimal temperature (30°C) and agitation speed (180 rpm), strain C1 produced on the same culture medium about 900 ± 50 μmol of IAA_equ_ per liter.

### *In Vitro* Rooting

Table 1 shows the effects of hormonal composition and concentration of compound applied into the rooting medium on stimulation of rooting from shoots of pear cv Dar Gazi. AR formation was strongly enhanced by the addition of metabolites secreted by *P. agglomerans* C1 (C1-sm) and to a lesser extent by IBA (Table 1). In the first experiment, two IAA_equ_ concentrations of secreted metabolites were tested: 1 (C1-sm) and 2 μM (2× C1-sm). Exometabolites from strain C1 were tested alone or in combination with IBA (1 μM), and experiments were repeated twice. Subsequently, with the aim of confirming the results, other trials were run and only C1-sm was used, alone or in combination with IBA. In all experiments, the positive role in the induction of AR clearly resulted when compared with IBA, confirming the observations made in the first two trials.

**Table 1:** Effect of IBA, C1-sm, and the combination of both on pear root production and *ex vitro* survival.

Treatment	Emergence time (days)		Rooted explants (%)	Roots per explant (n)	Main root length (mm)	*Ex vitro* survival (%)
	First root	Last root				
Control	-	-	0e	0e	-	0d
IBA (1 μM)	20 ± 4a	35 ± 1a	8d	1.0d^*^	7.9 ± 4.0b	83.4b
C1-sm (1 μM IAA_equ_)	7 ± 1c	14 ± 3c	58b	2.3 ± 0.3c	23.0 ± 2.7a	100a
2× C1-sm (2 μM IAA_equ_)	9 ± 2bc	ND	33c	2.8 ± 0.9b	9.1 ± 2.4b	ND
C1-sm + IBA	12 ± 1b	21 ± 5b	71a	4.0 ± 0.3a	10.2 ± 1.9b	66.6c

ND = not determined. Days until the first emergence of adventitious roots from the stem tissues; days occurred for the last adventitious root emergence from the stem tissues; the percentage of rooted explants; the number of roots per explant; the average of the longest root observed in each rooted explant; the percentage of *ex vitro* survival. Values represent the average ± se. Different letters indicate statistically significant differences between groups.

* Only one explant rooted for each repeated trial.

AR required a lot of time to emerge from the stem of microcuttings; a strong difference was in fact observed when the IBA and microbial IAA were added into the rooting medium both alone and in combination (Table 1). The formation of roots occurred in the microcuttings treated with C1-sm in a shorter time (7 ± 1 days after the treatment) than in microcuttings treated with C1-sm + IBA (12 ± 1 days after the treatment) and IBA alone (20 ± 4 days after the treatment; Table 1). Root emergence was not a synchronous event and occurred in a time span between 14 ± 3 and 35 ± 1 days (Table 1). Microcuttings treated with metabolites secreted by *P. agglomerans* C1 rooted earlier and for a shorter period than those treated with IBA alone (Table 1).

The number of ARs per explant induced by the combination of C1-sm and IBA (4.0 ± 0.3) was, respectively, 1.7‐ and 4-fold higher than that obtained using C1-sm and IBA alone (Table 1). Root formation was not observed in control experiments with plant growth medium: (1) without growth regulators; (2) with 16 mM tryptophan; or (3) with 250-fold diluted LB medium +4 mM tryptophan (Table 1).

As reported in Table 1, the use of C1-sm, alone or in combination with IBA, also stimulated root elongation. In particular, the length of the main root was 2.9-fold higher when the medium was supplemented with C1 exometabolites in place of IBA (Table 1).

**Figure 1:**
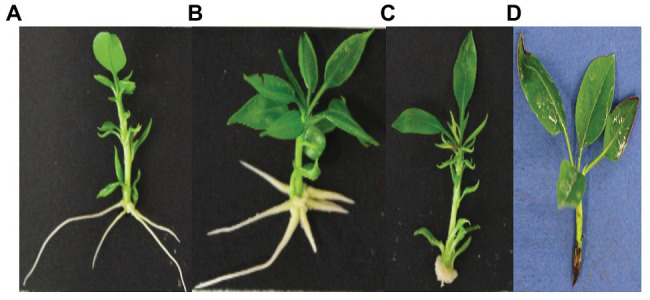
Adventitious root formation in microcuttings of *Pyrus communis* cv Dar Gazi cultured on MS medium added with C1-sm (1 μM IAA_equ_) **(A)**, a combination of C1-sm (1 μM IAA_equ_) and IBA (1 μM) **(B)**, and IBA (1 μM) **(C)** after 13 days of *in vitro* culture. Callus development was observed only when IBA was used alone or in combination with C1-sm **(B,C)**. No root formation was observed in control treatments with microcuttings cultured on MS medium without auxins **(D)**.

Interestingly, in the microcuttings where the ARs were induced by IBA or a combination of IBA and C1-sm, roots emerged from the undifferentiated callus tissue; on the contrary, in microcuttings treated with C1-sm, no callus formation was observed at the base of the shoot explants, without any apparent formation of callus tissues (Figure 1).

### Expression Analysis of Selected Auxin Signaling-Responsive Genes

#### Auxin Response Factor *PcARF6*, *PcARF8*, and *PcARF17* Genes

**Figure 2:**
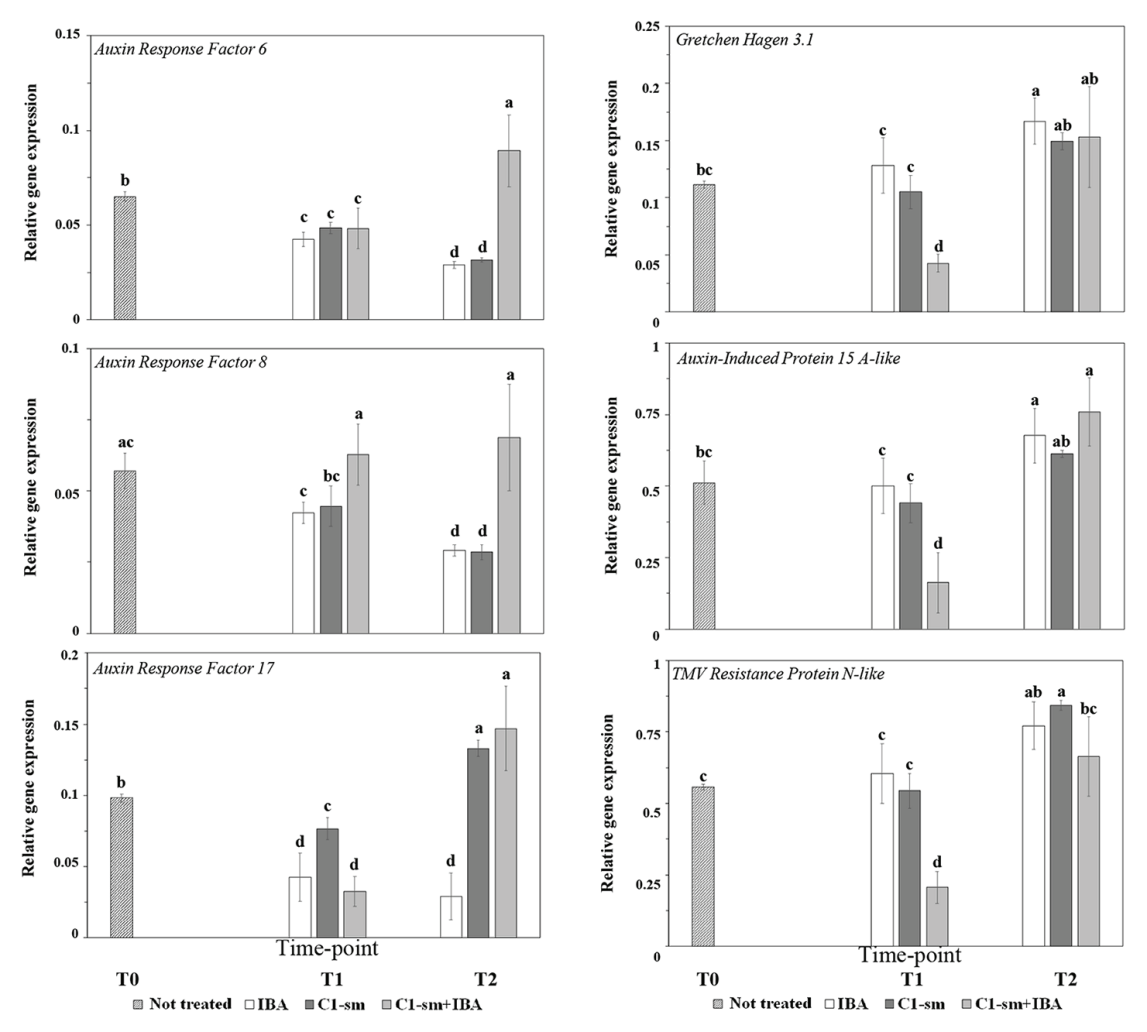
The expression pattern of selected *Pyrus communis* (Pc) genes responding to auxin signaling in microcuttings treated with IBA (1 μM), C1-sm (1 μM of IAA_equ_), and a combination of both, C1-sm (1 μM of IAA_equ_) + IBA (1 μM). The bar at time point T0 indicates expression in untreated control microcuttings, the bars at time point T1 indicate expression in microcuttings 4 days (IBA and C1-sm) or 6 days (C1-sm + IBA) after the treatment, the bars at time point T2 indicate expression at the initiation of root primordia. Relative expression of the target gene was normalized to the reference gene *Pc*Actin, as an internal control. Means are from three independent repeats; error bars show standard deviations. Different letters indicate statistically significant differences compared to control (T0) and between groups (*p* < 0.05).

Differential expressions of *PcARF6*, *PcARF8*, and *PcARF17* genes in pear microcuttings treated with IBA and C1-sm, alone or in combination with IBA, were investigated using real-time RT-PCR, and *Pc*Actin was used as a reference gene for normalization of target gene expression. The transcription analysis revealed that all these genes were downregulated by IBA (Figure 2). A similar effect was observed for *PcARF6* and *PcARF8* on microcuttings treated with C1-sm (Figure 2). In contrast, in cuttings treated with C1 exometabolites, the mRNA level of the *PcARF17* gene decreased during the dark period (T1) and increased at the initiation of root primordia (time point T2; Figure 2). Interestingly, the expression profiles of the three genes were quite different when microcuttings were treated with a combination of C1-sm and IBA, with *PcARF6* and *PcARF17* expressions being upregulated at the initiation of root primordia (T2) and *PcARF8* expression remaining constant across all time points (Figure 2).

#### Gretchen Haven 3.1 (*PcGH3.1*) Gene

*PcGH3.1* gene was upregulated at the initiation of root primordia (time point T2) in tissues treated with IBA (Figure 2). The expression profile of this gene was significantly different when tissues were treated with a combination of C1-sm and IBA, where a significant reduction of *PcGH3.1* mRNA level was observed 6 days after the treatment (T1; Figure 2). In contrast, no significant difference was observed comparing the relative expression of this gene in microcuttings treated with IBA or C1-sm alone (Figure 2).

#### Auxin-Induced Protein 15A-Like (*PcSAUR7*) Gene

The expression profile of *PcSAUR7* was similar to that observed for *PcGH3.1*, with a significant reduction of mRNA level at time point T1 on microcuttings treated with a combination of C1-sm and IBA and an increase in the mRNA level of the three genes between time points T1 and T2 (Figure 2).

#### TMV Resistance Protein N-Like (*PcTMV*) Gene

The expression profile of *PcTMV* on microcuttings treated with IBA and C1-sm alone and in combination followed the same expression trend observed for *PcGH3.1* and *PcSAUR7* under the same conditions (Figure 2).

## Discussion

In previous work, we demonstrated that the production of IAA and IAA-related compounds by *P. agglomerans* strain C1 is tryptophan dependent and is affected by several parameters, including the medium composition, the carbon source, the physiological state of the cells, and the induction timing (Luziatelli et al., 2020b). Usually, standard experiments with PGP bacteria are designed to obtain the maximum auxin production, and to avoid inhibitory effects on the plant growth, culture filtrates containing a higher IAA_equ_ titer are extensively diluted before use as plant biostimulants. Although, in general, bacterial strains exhibiting higher volumetric productivity of a specific metabolite are preferred for large-scale production, in this context, extensive dilution of the culture filtrate can be detrimental to evaluating the synergistic effects that metabolites secreted by IAA/auxin-producing bacteria can have on transport and turnover of exogenous and endogenous auxin in plants (Luziatelli et al., 2020b). In this study, we demonstrated that the auxin production by *P. agglomerans* strain C1 can be modulated (until one-third of the maximum) by lowering the growth temperature and the aeration of the liquid culture. The cultivation under suboptimal conditions alters the balance between auxin and other metabolites constitutively produced by strain C1 and allows the use of its spent growth medium at a higher strength.

In microcuttings of *P. communis* L. cv Dar Gazi cultured into rooting medium enriched with exometabolites from auxin-producing cultures of *P. agglomerans* C1, AR emergence occurred earlier than in cuttings that cultured into IBA-enriched rooting medium. In addition, in (C1-sm)-treated shoots (Figure 1), all roots were located at the side of the stem, indicating that the primordia of AR directly develop from the cells neighboring vascular tissues and the subsequent elongated root directly emerged from the epidermal tissue of side of the stem, just above the basal pruned zone (de Klerk et al., 1999; Lakehal and Bellini, 2019). The direct rooting formation resulted independently from the concentration of C1-sm used, and when the medium was enriched with IBA, either alone or in combination with C1-sm, another contrasting morphological scenario was evident. As a first step, a cell division forming callus tissue was observed, and then AR was observed, probably, after the reprogramming of the AR founder cell (Bustillo-Avendaño et al., 2018). Our data are also in agreement with the observations demonstrating that the auxin transcriptional networks involved in both direct and indirect *de novo* root regeneration (DNDR) and callus formation in the model plant *Arabidopsis* partially share the same genetic pathway (reviewed in Jing et al., 2020).

It is already known that in cuttings of woody species, wounding can stimulate callus formation and AR primordia can emerge from the cells of this undifferentiated tissue (Li et al., 2009; Xu, 2018). In microcuttings of the apple tree, the AR initiation occurred from cells of the cambial zone located between the two vascular tissues of the stem, without the formation of callus, and the root emerged outside the stem 15 days after the treatment with IBA (Naija et al., 2008). Furthermore, the time of root emergence outside the stem and the diverse root morphology in microcuttings treated with C1-sm and IBA reinforced the hypothesis that the AR phenotype is regulated differently by the two treatments.

To evaluate the synergistic effect of auxin and other metabolites produced by strain C1, experiments were carried out by using two different amounts of growth medium, and interestingly, better results in terms of percentage of rooted explants and the average length of the main root were obtained with C1-sm at the lower concentration (1 μM IAA_equ_; Table 1). It is noteworthy that, upon a combination of C1-sm and IBA, we observed a 40% reduction in the timing of AR emergence compared to IBA, as well as a ninefold increase in the percentage of rooted explants and a fourfold increase in the number of roots per explant, while the length of the main root increased compared to IBA, but not to the same extent when using C1-sm alone (Table 1). These results confirm our hypothesis that the balance between auxin and other biostimulant molecules produced by strain C1, as well as the appropriate gradient generated by the amount of exogenous auxin that is used, plays a critical role in determining the performance of the biostimulant during the rooting stage of micropropagation, improving the efficiency of the nursery industry. In this study, IBA was applied at a concentration (1 μM) less than fivefold lower than the one which is usually used in the pear rooting medium (Reed et al., 2012), so it is not surprising that the addition of auxins present in C1-sm has a positive effect on AR formation. At the same time, the reduction in the percentage of rooted explants and in the length of the main root, which was observed when IBA was added to C1-sm, indicates that, when strain C1 metabolites are present at a certain concentration, an excess of auxins can inhibit the formation and the elongation of the adventitious roots (Table 1). The differences in the root architecture that are shown in Figure 1 clearly indicate that the metabolites produced by *P. agglomerans* C1 not only accelerate the AR formation when auxins are present but also increase the number and length of AR primordia that develop from stems, modifying the response of the pear plant to exogenous auxins.

In recent work, Lotfi et al. (2019) demonstrated that *trans*-cinnamic acid, a phenylpropenoic compound produced by the microbial deamination of phenylalanine (Hazir et al., 2017), efficiently induces AR formation in shoots of pear cv Arbi. It is noteworthy that *P. agglomerans* C1 has genes encoding enzymes (e.g., a CAT-catalase, ec 1.11.1.6) that are involved in the biosynthesis of cinnamic-related compounds from intermediates of the tryptophan pathway, and we have evidence that strain C1 produces cinnamates as well as peptides and cyclopeptides that can crosstalk with auxin (Luziatelli et al., 2020b). These compounds may likely play a possible regulative role in plant gene expression through epigenetic mechanisms, such as DNA methylation, histone modification (acetylation, methylation, and phosphorylation), and miRNA activation, as it already emerged in humans (Zhu et al., 2016; Frolinger et al. 2018; Arora et al., 2019; Carlos-Reyes et al., 2019; Patnaik and Anupriya, 2019). These mechanisms likely activate many regulatory pathways generating a response, in parallel to the pathway activated by auxin compounds, and thus establish a synergistic action. It is known, in fact, that polyphenols, such as phloroglucinol, caffeic acid, chlorogenic acid, and ferulic acid, have a synergistic action, together with auxins, as mediators of root formation from cuttings (James and Thurbon, 1981; Zimmerman and Broome, 1981; Wilson and Van Staden 1990; Wang, 1991; Nandi et al., 1996; De Klerk et al., 2011).

To gain insights into the molecular mechanisms elicited by strain C1 exometabolites, we analyzed the expression profile of three auxin response factor (*ARF*) genes, which play a key role in the AR initiation process and in auxin homeostasis. Results reported in Figure 2 indicated that these genes were differentially modulated in microcutting tissues treated with C1-sm or IBA. The temporal expression patterns of *PcARF6* and *PcARF8* were similar, while significant differences were observed in the temporal transcription pattern and in the steady-state transcript level of *PcARF17* (Figure 2). The latter gene was overexpressed in microcuttings treated with C1-sm during the rooting phases examined in this work (Figure 2). Auxin homeostasis plays a key role in the AR formation: the level of free auxins, in the model plant *Arabidopsis thaliana*, controls the expression of the *ARF*s, *AtARF6*, *AtARF8*, and *AtARF17*, that activate a signaling cascade to lead to the AR initiation in the target cells until the emergence of root primordium (Gutierrez et al., 2012; Guan et al., 2015). These genes are also under the control of light and, when cuttings of *A. thaliana* are transferred from darkness to light, *ARF6* and *ARF8* are upregulated, while *ARF17* is downregulated (Fattorini et al., 2018). In our experimental conditions, in microcuttings treated with IBA or C1-sm, the orthologous *PcARF6* and *PcARF8* genes were downregulated, and at each time point, no significant difference in transcript levels of these genes was observed (Figure 2). In contrast, when IBA was used in combination with C1-sm, *PcARF6* was upregulated at sampling point T2, while the mRNA level of *PcARF8* was stable across the three time points (Figure 2). Taken together, these results indicate that the addition of C1-sm to IBA determines a differential transcriptional or posttranscriptional regulation of *PcARF6* and *PcARF8* genes that is probably related to the different morphological and temporal responses elicited by IBA and C1-sm + IBA (Table 1; Figure 1). The temporal expression profiles of these *ARF* genes in cv Dar Gazi differ from those reported in the literature, but they appear to be consistent with data obtained *in vitro* with apple microcuttings, in which expressions of *ARF6* and *ARF8* were not observed during AR formation (Li et al., 2016). The same authors also demonstrated that, in apple microcuttings, a treatment with IBA determined an upregulation of *ARF9* gene and a downregulation of *ARF1* and *ARF11*. It is worth mentioning that, although no significant difference was found in the temporal expression patterns of *PcARF6* and *PcARF8* in microcuttings treated with IBA (1 μM) or C1-sm (1 μM IAA_equ_), the different timing of root primordium initiation at equal auxin molarity (7 and 20 days, respectively; Table 1) suggests that the auxin signal transduction is more effective and faster when target cells were treated with strain C1 exometabolites. This hypothesis is supported by the observation that, regardless of the speed of the process, at the AR emergence (time point T2), a significant reduction in the steady-state transcript levels of *PcARF6* and *PcARF8* was observed in both IBA‐ and C1-sm-treated microcuttings. It is reasonable to assume that the downregulation of *PcARF6* and *PcARF8* observed at time point T2 is related to the low concentration of auxin (1 μM) that was used in this study. According to the auxin signaling model proposed by Guilfoyle and Hagen (2007), the expression of these genes should be downregulated when auxin concentration is below a threshold level because ARF6 and ARF8 proteins act as transcriptional activators of *GH3* genes, which encode auxin adenylating enzymes. The same model can also explain results obtained analyzing the expression of the *PcARF17* gene at time point T1, which indicated downregulation of this gene independently of treatment (Figure 2). In *A. thaliana*, it has been demonstrated that ARF17 is a negative regulator of AR formation and is an antagonist of *AtARF6* and *AtARF8* genes (Gutierrez et al., 2009; Villacorta-Martín et al., 2015). So it can be suggested that by changing the concentration of ARF17 protein and modifying the balance among ARF transcriptional activators and repressors, the AR formation promoted by ARF6 and ARF8 can be promoted. Interestingly, at time point T1, the steady-state transcript level of *PcARF17* significantly increased when replacing IBA with C1-sm, and independently from IBA, this gene was upregulated in the presence of strain C1 exometabolites (Figure 2). In accordance with the observations reported in the literature (Gutierrez et al., 2012; Gleeson et al., 2014; Ruedell et al., 2015), it can be postulated that the upregulation of *PcAFR17* can explain, at least in part, the altered adventitious root phenotype of C1-sm-treated microcuttings.

C1-sm may contain metabolites that likely behave as co-inducers and/or inhibitors of AR formation. The higher expressions at time point T2 of *PcAFR17* and all *PcARF* genes when C1-sm was used alone or in combination with IBA, respectively (Figure 2), may imply that C1-sm contains molecules which interfere with auxin homeostasis and, in synergy with auxin, accelerate the AR formation process. In this context, the regulatory effect that the metabolites secreted by *P. agglomerans* C1 have on the transcriptional and posttranscriptional control of the *PcAFR17* gene plays a central role in the modification of the root traits and temporal patterns that occur in Dar Gazi.

Interestingly, independent of treatment, the temporal expression patterns of *PcGH3.1*, *PcSAUR7*, and *PcTMV* were similar (Figure 2). All three genes were upregulated at time point T2 when IBA or C1-sm was used alone and were downregulated at time point T1 when IBA was used in combination with C1-sm (Figure 2). These results are in accordance with data obtained on apple microcuttings, which indicate that the expressions of these genes are stimulated by a reduction in the concentration of free auxin (Li et al., 2019). At the same time, the downregulation of the three genes observed at time point T1 after treatment with C1-sm + IBA can be due to the auxin initial concentration that is doubled compared to the conditions in which IBA and C1-sm are used alone.

Genes *Gretchen Hagen 3* (*GH3*) is a family of genes involved in processes of the conjugation of auxins and regulates free auxin content (Ludwig-Müller, 2011; Westfall et al., 2016). The *GH3.1* gene encodes an auxin-conjugating enzyme involved in modulating the level of free auxin (Druege et al., 2016).

The trend of *PcGH3.1* gene expression that was observed in the different treatments at time point T2 (Figure 2) confirms the hypothesis by Druege et al. (2016). According to them, as soon as the rooting induction is completed, the level of free auxin must decrease in order to avoid an uncontrolled induction of root primordia and inhibit the development of AR previously developed. The minor expression of *PcGH3.1* that is observed at time point T1 in tissues treated with C1-sm + IBA may imply that auxin remains in a free form for a longer time when exometabolites from strain C1 are present and thus induces more efficiently the AR formation.

The stability of the auxin signal depends on the type of auxin used in the culture medium. It is known, in fact, that IAA degrades very quickly when it undergoes high temperatures, air, and light (Yamakawa et al., 1979), while IBA, which is a synthetic hormone, is more stable (Robbins et al., 1988). It may thus be hypothesized that auxins present in C1-sm may degrade some days after treatment and, therefore, do not generate unbalances in plant hormone homeostasis when C1-sm and IBA are added together. However, it should be emphasized that in this study, we reduced the hormone concentration, which is usually 5 μM (Jan et al., 2015), up to 1 μM, in order to reduce the risk of uncontrolled accumulation of auxin.

The auxin-induced protein 15A-like encoding gene from *P. communis* is homologous to the “small auxin upregulated RNA” *SAUR7* gene from *A. thaliana*. *SAUR7* encodes a protein involved in an early response of the auxin stimulus and can be used as a marker gene to evaluate the efficiency of auxin treatment (Goda et al., 2004). Instead, the pear gene *PcTMV*, orthologous to the *AtTMV* gene, encodes a resistance protein, TIR-NBS-LRR, belonging to the family of response proteins to pathogens R and can be used as a sensor of the response to exogenous auxin and high-light stress (Huang et al., 2019). In microcuttings treated with C1-sm, the steady-state transcript level of both genes in the different rooting phases was similar to that observed in IBA-treated microcuttings, confirming that *P. agglomerans* C1 metabolites impact the timing of the auxin turnover rather than the accumulation of auxin.

In conclusion, the results obtained in this study show that the metabolites secreted by *P. agglomerans* C1 contain molecules that act in synergy with auxins and maintain an optimal gradient of this hormone, which positively affect the temporal pattern of *de novo* root formation, as well as root morphology and efficiency. Identification and characterization of these molecules will be useful to investigate the underlying mechanisms leading to the modification of AR development.

## Data Availability Statement

The genome sequence of *P. agglomerans* C1 is available under NCBI with genome assembly number ASM975988v1.

## Author Contributions

FL, LG, AF, RM, and MR contributed to the conception and design of the study. FL, LG, AF, CS, RM, and MR contributed to defining the methodology. FL, LG, AF, RM, and MR contributed to data validation. FL, AF, LG, GM, CS, FM, RM, and MR contributed to the investigation. FL, RM, and MR wrote the first draft of the manuscript. FL, FM, RM, and MR wrote and edited the final version of the manuscript. All authors have read and agreed to the published version of the manuscript. All authors contributed to the article and approved the submitted version.

### Conflict of Interest

The authors declare that the research was conducted in the absence of any commercial or financial relationships that could be construed as a potential conflict of interest.

## Footnotes

^1^http://www.fao.org/faostat/en/#data/QC
